# Tinnitus and Its Relation to Depression, Anxiety, and Stress—A Population-Based Cohort Study

**DOI:** 10.3390/jcm12031169

**Published:** 2023-02-01

**Authors:** Berit Hackenberg, Julia Döge, Karoline O’Brien, Andrea Bohnert, Karl J. Lackner, Manfred E. Beutel, Matthias Michal, Thomas Münzel, Philipp S. Wild, Norbert Pfeiffer, Andreas Schulz, Irene Schmidtmann, Christoph Matthias, Katharina Bahr

**Affiliations:** 1 Department of Otorhinolaryngology, University Medical Center Mainz, 55131 Mainz, Germany; 2 Institute for Clinical Chemistry and Laboratory Medicine, University Medical Center Mainz, 55131 Mainz, Germany; 3 Department of Psychosomatic Medicine and Psychotherapy, University Medical Center Mainz, 55131 Mainz, Germany; 4 Department of Cardiology—Cardiology I, University Medical Center Mainz, 55131 Mainz, Germany; 5 Preventive Cardiology and Preventive Medicine—Department of Cardiology, University Medical Center Mainz, 55131 Mainz, Germany; 6 Center for Thrombosis and Hemostasis, University Medical Center Mainz, 55131 Mainz, Germany; 7 DZHK (German Center for Cardiovascular Research), Partner Site Rhine-Main, 55131 Mainz, Germany; 8 Institute of Molecular Biology (IMB), 55128 Mainz, Germany; 9 Department of Ophthalmology, University Medical Center Mainz, 55131 Mainz, Germany; 10 Institute of Medical Biostatistics, Epidemiology and Informatics, University Medical Center Mainz, 55131 Mainz, Germany

**Keywords:** tinnitus, depression, anxiety, cohort study

## Abstract

Tinnitus is a common symptom reported in otolaryngologic practice. Although the pathophysiology of tinnitus has not been fully understood, clinical studies suggest that psychological symptoms of depression, anxiety, and somatization are increased in tinnitus patients. However, patients seeking medical treatment for tinnitus may be especially vulnerable. Population-based studies reporting on the association between tinnitus and psychological distress are still lacking. The aim of this study was to investigate the correlation of tinnitus with depression, anxiety, or somatization in a large population-based cohort. The Gutenberg Health Study is a population-based cohort study. Participants were asked about the occurrence of tinnitus (yes/no) and how much they were bothered by it. In addition, they completed the PHQ-9, GAD-7, and SSS-8 questionnaires to assess depressive symptoms, anxiety, and somatic symptom disorders. A total of 8539 participants were included in the study cohort. Tinnitus prevalence was 28.0% (2387). The prevalence of depression/anxiety/somatic symptom disorders was significantly higher among participants with tinnitus than among participants without tinnitus (7.9%/5.4%/40.4% participants with tinnitus vs. 4.6%/3.3%/26.9% participants without tinnitus, *p*-value < 0.0001). Logistic regression results showed that participants with tinnitus were more likely to suffer from depression (OR = 2.033, 95% CI [1.584; 2.601], *p*-value < 0.0001), anxiety (OR = 1.841, 95% CI [1.228; 2.728], *p*-value = 0.0027), or somatic symptom disorders (OR = 2.057, 95% CI [1.799; 2.352], *p*-value < 0.0001). Symptoms of depression, anxiety, and somatic symptom disorders were increased in participants with tinnitus. This must be taken into account when treating these patients.

## 1. Introduction

Tinnitus is defined as a sound perceived without stimulus from an external acoustic source [1]. The prevalence ranges from 5 to 43% worldwide and from 9 to 28% in Europe [2,3]. This prevalence appears to increase with age, and males are more commonly affected [2,4]. The wide range of prevalence data may be due to the lack of a common definition of tinnitus in research [4].

In clinical practice, a higher rate of psychological comorbidities is observed in tinnitus patients. Prevalence rates for depressive disorders in tinnitus patients range from 14 to 80% [5,6]. In addition, a review on tinnitus and depression included 20 studies and found 18 studies with a positive correlation between tinnitus and depression [7]. Most of these studies were cross-sectional studies and only one study included more than 500 patients (*n* = 1275) [8]. 

Mental disorders themselves have a high prevalence in Germany. In 2019/2020, clinically significant depressive symptoms were reported by 8.3% of the population. Females were affected more frequently than males (8.8% vs. 7.5%). Depressive symptoms decreased with age, reaching a minimum prevalence of 5.0% in the 65- to 79-year-old age group [9]. The prevalence of anxiety is 15.3% in Germany. Once again, the prevalence is higher in females (21.4% in females and 9.3% in males) [10]. In addition, somatic symptom disorders occur in 9.11% of the German population. Females were affected significantly more than males [11]. With a high prevalence of both tinnitus and mental disorders, it is difficult to distinguish correlation from coincidence. It remains unclear whether depression is a factor that exacerbates tinnitus, whether tinnitus is a predisposing factor for depression, or whether both occur as comorbidities [7]. Most studies reporting on tinnitus and depression are limited by the fact that the study population consists of patients seeking care for tinnitus or depression. This may result in a selection bias of unknown magnitude [12]. 

The pathophysiological pathways of tinnitus development are not fully understood [13]. Tinnitus is hypothesized to arise due to audiological and somatosensory dysfunction. Cochlear lesions due to hearing loss are hypothesized to cause this imbalance [14]. Hearing loss can reduce nerval activity and downregulate cortical inhibition. This may lead to hyperexcitability in the auditory cortex and could form the basis of tinnitus formation [15]. Therefore, it seems reasonable to consider hearing impairment as a risk factor for tinnitus [1]. Cognitive-emotional and psychological factors are hypothesized to play an additional role in the exacerbation of an occasional tinnitus into a chronic and bothersome one [16,17]. 

This study adds to the literature by reporting the association of tinnitus, depression, anxiety, somatic symptom disorders, and hearing impairment tested for by pure-tone audiometry in a large population-based cohort. Thereby, this study is the first to investigate this relationship in the general population rather than in patients.

## 2. Materials and Methods

The Gutenberg Health Study (GHS) is a large population-based cohort study. It was initiated in 2007 at Mainz University Hospital in Germany and includes participants drawn at random from the residents’ registration office. Therefore, the study population is representative of the population of Mainz and the district of Mainz-Bingen. The baseline cohort was examined from 2007 to 2012 and included 15,000 participants (core cohort). While a computer-assisted telephone interview was conducted with all participants after 5 years, all participants in the core cohort were invited to visit the study site again for the 10-year follow-up. At this 10-year follow-up (from 2017 to 2020), an otologic testing was included in the study design. In addition to the core cohort, new participants for ages 25 to 44 years (young cohort, *n* = 4000) and 75 to 85 years (senior cohort, *n* = 1000) were recruited. All three cohorts will be followed up in 10-year increments through 2027 [18]. A detailed description of the study design can be found elsewhere [19]. 

In the 10-year follow-up, from 2017 to 2020, participants completed a questionnaire about their auditory quality of life: “Do you suffer from ringing in the ears (tinnitus)?” (yes/no) and “How much do you feel burdened by it?” (six-level scale, ranging from 1 = “little stressful” to 6 = “extremely stressful”). Furthermore, participants underwent audiologic testing using pure-tone audiometry for air- and bone-conduction [20]. 

Participants completed the following psychometric questionnaires in order to evaluate their mental health quality of life: Patient Health Questionnaire-9 (PHQ-9), Generalized Anxiety Disorder Scale-7 (GAD-7), and Somatic Symptom Scale-8 (SSS-8). The PHQ-9 is a standardized instrument that measures depressive symptoms based on the DSM-IV criteria (Diagnostic and Statistical Manual of Mental Disorders) [21]. It assesses symptoms that have occurred within the past 2 weeks. A total score of >10 (of 27) was considered as a depressive mood [22]. The GAD-7 is a validated instrument for measuring anxiety in the general population [23]. The sum score ranges from 0 to 21, and a GAD-7 score of >10 (indicating moderate or severe anxiety) was considered as a positive score for anxiety. As an abbreviated version of the Patient Health Questionnaire (PHQ-15), the SSS-8 was constructed and validated to identify somatic symptom burden [24]. Eight items measure subjective impairment related to non-specific physical symptoms, such as stomach or bowel problems, headaches or trouble sleeping. A sum score of eight or higher was considered as suspicious of a somatic symptom disorder. All three instruments have been used in tinnitus patients [25,26,27,28]. 

Exclusion criteria for the GHS study were disabilities that prevented participants from visiting the study site. In addition, participants without sufficient knowledge of the German language had to be excluded since questionnaires were only provided in German. Furthermore, in this evaluation, participants had to be retrospectively excluded due to missing data on tinnitus (yes/no). Prevalence rates of tinnitus (yes/no), depressive symptoms (PHQ-9 > 10), anxiety (GAD-7 > 10), and somatic symptom disorder (SSS-8 > 8) were calculated for the study sample. The prevalence rates were weighed to the European Standard Population 2013 (ESP) and to the German Standard Population 2021 (GSP). 

Hearing impairment was calculated based on the revised WHO classification of hearing impairment (hearing threshold in the better hearing ear in decibles (dB), average across 0.5/1/2/4 kHz) [29]. Three groups were formed for statistical analyses: No hearing impairment (<20 dB), mild to moderately severe hearing impairment (20–64.9 dB), and severe to complete hearing impairment (>65 dB). Spearman’s rank correlation coefficients were calculated to measure the degree of association between tinnitus burden (1–6) and scores in PHQ-9, GAD-7, and SSS-8. A multiple logistic regression model tested the prediction of tinnitus likelihood as a function of hearing loss, depressive symptoms, anxiety, and somatic symptom disorder. All statistical analyses were completed in R version 4.1.0 (18 May 2021): R Core Team (2021). 

The study was approved by the local institutional review board (Ethics Commission of the State Chamber of Physicians of Rhineland-Palatine, reference no. 837.020.07) and was conducted in full compliance with the Declaration of Helsinki. Written informed consent was obtained from all subjects before participation in the study.

## 3. Results

The GHS cohort (core, young, and senior) comprised a total of 20,000 participants. For the 10-year follow-up, 9752 participants attended the study site. Of these, 1213 (12.4%) had to be excluded for missing data on tinnitus (y/n). Therefore, 8539 participants could be included in the study cohort. In this cohort, 4362 participants were male (51.1%) and 4177 were female (48.9%). The mean age was 60.7 years, with females averaging slightly, but significantly, younger than males (females: 59.9 years, males: 61.4 years). Tinnitus point prevalence was 28.0% (*n* = 2387). Tinnitus occurred more frequently in males than in females (males: 31.7%, females: 24.1%, *p* < 0.0001). Females seemed more burdened by their tinnitus than males, although this difference was statistically not significant (mean burden: All: 2.39 (±1.33), females: 2.44 (±1.34), males: 2.35 (±1.33), *p*-value = 0.11). A total of 5.5% of all participants reported clinically significant depressive symptoms (PHQ-9 > 10). Females were more frequently affected than males (females: 6.7%, males: 4.4%, *p* < 0.0001). Anxiety (GAD-7 > 10) had a prevalence of 3.9% (*n* = 169) in our cohort. Females had a higher prevalence than males (females: 5.3%, males: 2.6%, *p* < 0.0001). The prevalence of somatic symptom disorders was 30.6% (*n* = 2584). Once again, females were more frequently affected than males (females: 36.6%, males: 25.0%, *p* < 0.0001) (see Table 1). 

**Figure 1:**
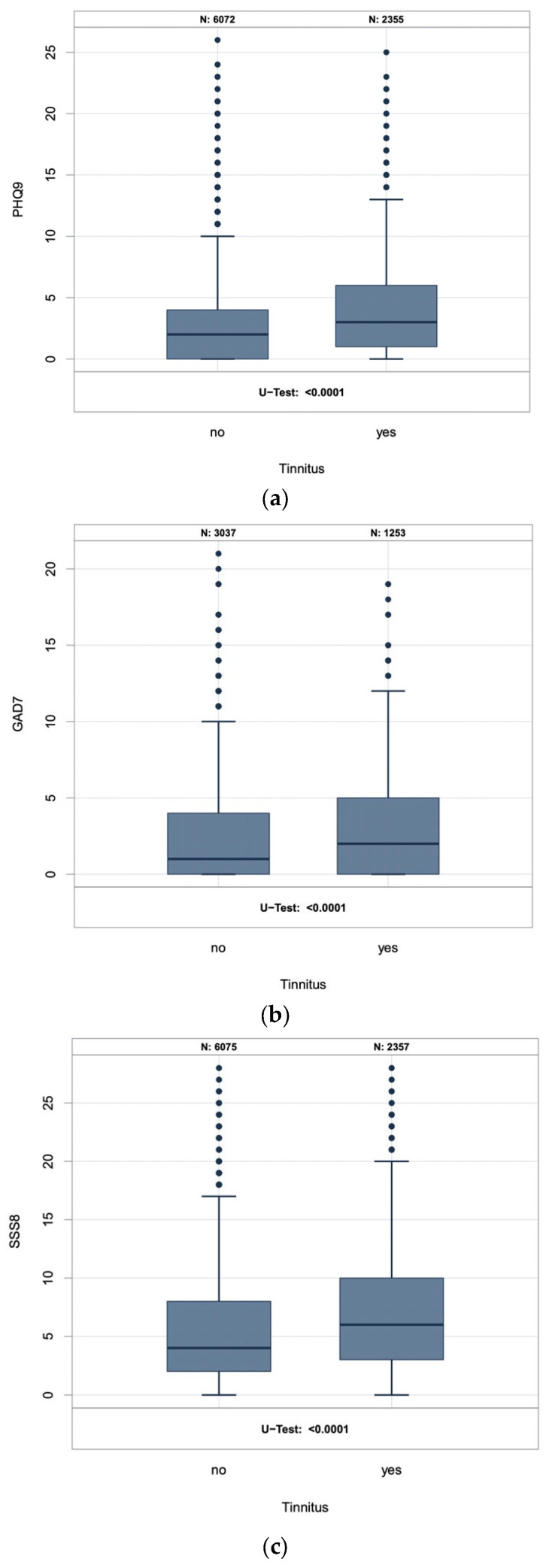
Sum score of the (**a**) PHQ-9, (**b**) GAD-7, and (**c**) SSS-8 in participants with and without tinnitus.

In our study cohort, the prevalence of depression, anxiety, and somatic symptom disorders was higher among participants with tinnitus than among participants without tinnitus. Clinically significant somatic stress symptoms had the highest prevalence among tinnitus participants at 40.4% (95% CI [38.4%; 42.4%]), followed by depression at 7.9% (95% CI [6.9%; 9.1%]), and anxiety at 5.4% (95% CI [4.3%; 6.9%]) (see Table 2). When these results are weighed to the ESP, somatic symptom disorders show a prevalence of 41.0% (95% CI [38.9%; 43.1%]) among tinnitus participants, while depression and anxiety show a prevalence of 9.5% (95% CI [8.3%; 10.9%]) and 8.8% (95% CI [7.6%; 10.1%]), respectively. Somatic symptom disorders weighed to the GSP showed an even higher prevalence of 41.6% (95% CI [39.5%; 43.7%]) among tinnitus participants, followed by 9.3% (95% CI [8.1%; 10.6%]) prevalence of depression and a 5.6% (95% CI [4.3%; 7.3%]) prevalence of anxiety among tinnitus participants. In all cases, tinnitus participants were more frequently affected than participants without tinnitus. Mean scores on the PHQ-9, GAD-7, and SSS-8 were higher in participants with tinnitus than in those without tinnitus (Figure 1). 

A total of 51.4% (2664) of the study participants had mild to moderately severe hearing impairment. In addition, 2.0% (104) had severe to complete hearing impairment and 46.6% had no hearing impairment (see Table 3). Hearing impairment was more prevalent in participants with tinnitus than in participants without tinnitus. The majority of tinnitus participants (59.3%) were found to have mild to moderately severe hearing loss, compared with 48.6% of participants without tinnitus. This remained unchanged when the results were generalized to the ESP and GSP (see Table 4). Both ESP and GSP showed a distribution of age groups with a younger average than the study cohort. 

**Figure 2:**
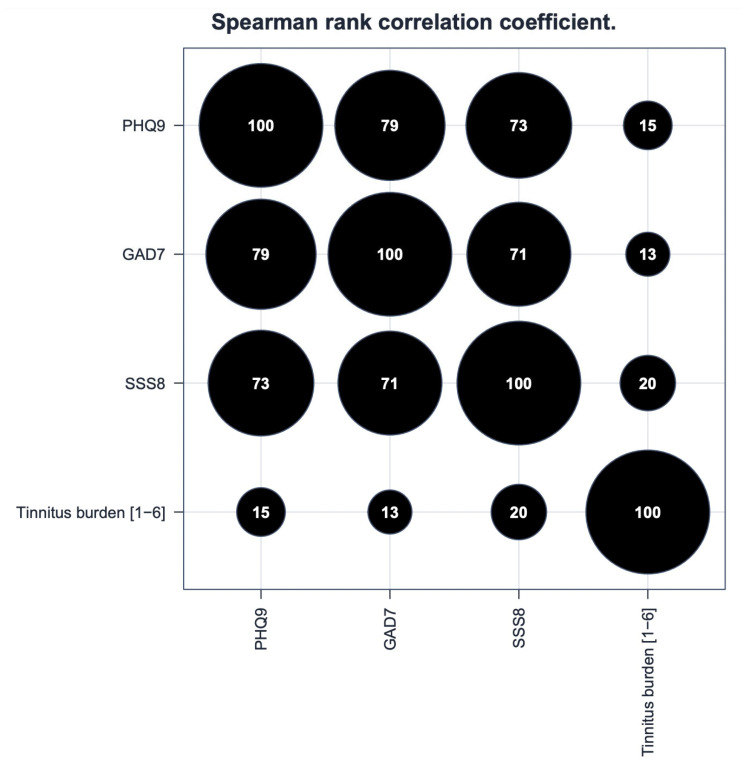
Spearman’s rank correlation coefficient between the sum scores of PHQ-9, GAD-7, SSS-8, and tinnitus burden (1–6) (×100).

Spearman’s rank correlation coefficient showed a positive correlation between tinnitus burden and scores in PHQ-9 (correlation: 0.15), GAD-7 (correlation: 0.13), and SSS-8 (correlation: 0.2) (Figure 2). The applied standardized psychometric questionnaires showed the highest internal correlation of scores between PHQ-9 and GAD-7 (correlation: 0.79), followed by PHQ-9 and SSS-8 (correlation: 0.73), and GAD-7 and SSS-8 (correlation: 0.71).

Logistic regression results showed that participants with tinnitus were more likely to suffer from depression (OR = 2.033, 95% CI [1.584; 2.601], *p*-value < 0.0001), anxiety (OR = 1.841, 95% CI [1.228; 2.728], *p*-value = 0.0027), or somatic symptom disorders (OR = 2.057, 95% CI [1.799; 2.352], *p*-value < 0.0001). Including all independent variables in a model, the likelihood of severe to complete hearing loss (>65 dB) was highest in tinnitus participants (OR = 4.819, 95% CI [2.763; 8.445], *p*-value < 0.0001) followed by somatic symptom disorders (OR = 1.836, 95% CI [1.506; 2.236], *p*-value < 0.0001). With an increasing prevalence of tinnitus with age, the OR for tinnitus in 10-year increments of age was 1.118 (95% CI [1.080; 1.159], *p*-value < 0.0001). After adjusting for hearing impairment, age was no longer relevant (mild to moderately severe hearing impairment: OR = 1.733, 95% CI [1.384; 2.174], *p*-value < 0.0001; severe to complete hearing impairment: OR = 4.819, 95% CI [2.763; 8.445], *p*-value < 0.0001).

In addition, the logistic regression model showed that mild to moderately severe hearing loss (as opposed to normal hearing) increased the likelihood of tinnitus significantly (OR = 1.648, 95% CI [1.405; 1.936], *p*-value < 0.0001). When comparing severe to complete hearing loss with normal hearing, the odds ratio of tinnitus was even bigger (OR = 4.806, 95% CI [3.162; 7.337], *p*-value < 0.0001). This influence of hearing loss on tinnitus did not change after adjusting for depression, anxiety, or somatic symptom disorders separately or for all three combined (see Table 5). 

## 4. Discussion

The purpose of this study was to report the prevalence and correlation of tinnitus, depression, anxiety, and somatic symptom disorders in the general population. The main findings of this study were higher prevalence rates of depression, anxiety, and somatic symptom disorders in participants with tinnitus compared with participants without tinnitus. Hearing loss, although known to be a risk factor for tinnitus, did not significantly affect this relationship.

Our study showed a significantly increased prevalence of depression among the population-based study sample. This compares well with previous literature. A recent study by Park et al. examined 10,979 participants. They asked about tinnitus (y/n) and used the PHQ-9 for depressive symptoms. Moreover, they rated a sum score of >10 on the PHQ-9 as depression, making the results comparable to our study [26]. Reported rates of depression were slightly lower than our results, but also showed significantly higher rates of depression among participants with tinnitus. The odds ratio (OR) of depression in participants with tinnitus was 1.78, which was slightly lower than our OR, but still within our reported confidence interval of 1.584 to 2.601. 

The PHQ-9 and GAD-7 have also been used in studies including otolaryngologic patients [25,27]. Al-Rawashdeh et al. enrolled 1328 adult otolaryngologic out-patients [25]. Symptoms and/or disorders were recorded in 727 patients, of whom 59 (8.1%) presented with tinnitus. Tinnitus patients had statistically significant higher scores on the PHQ-9 and GAD-7, which is in line with our study results. The OR of depression in tinnitus patients was 2.716, which was higher than the OR found in our study cohort. This could be due to the fact that Al-Rawashdah et al. included patients who sought medical care. With depression severity increasing with tinnitus burden, a higher prevalence of depression must be assumed in severely affected individuals. Moreover, Williams et al. evaluated tinnitus patients by using the PHQ-9 and GAD-7 [27]. They were able to include 107 patients and found a median score of 5 on PHQ-9 and of 4 on the GAD-7. The prevalence of depression (defined by PHQ-9 > 10) ranged from 15 to 36% depending on the subgroup (patients with pulsatile tinnitus vs. non-pulsatile tinnitus). Anxiety (GAD-7 > 10) had a prevalence ranging from 20 to 51% depending on the subgroup. Spearman’s rank correlation showed a positive correlation between PHQ-9/GAD-7 sum scores and Tinnitus Handicap Index scores, which is in line with our results of tinnitus burden correlating with psychological symptom severity. The prevalence rates of depression/anxiety in tinnitus patients and the sum scores were higher than those found in our study cohort. This may be due to the fact that Williams et al. included patients who sought care for their tinnitus. Higher burden and thus higher suffering could be assumed and would explain these differences. 

In a study by Boecking et al., the SSS-8 was used in tinnitus patients [28]. They found mean sum scores of 12.96 in females and of 10.09 in males. These scores are higher than in our sample, although we also found a significantly higher prevalence of somatic symptom disorders in females than in males. 

With the exception of the study by Park et al., studies have applied the PHQ-9, GAD-7, and SSS-8 to patients seeking treatment for tinnitus. These studies are limited by a selection bias of a population likely to have higher levels of distress. The strength of this study is its population-based design. This allowed us to include participants with tinnitus regardless of associated tinnitus burden or psychological distress. Furthermore, we evaluated participants without tinnitus as controls. 

A particularly striking result of our study is that somatic symptom disorders were the most common among tinnitus participants (40.4%). Somatic symptom disorders are defined by one or more somatic symptoms that are disproportionally distressing to the patient or even disrupt their daily life. Studies attempting to decipher the pathophysiology underlying the occurrence of tinnitus locate its origin in aberrant neural activities in the anterior cingular cortex, the anterior insula, and amygdala [30,31,32]. These neuroanatomic correlates also appear to be actively involved in chronic pain syndromes and somatic symptom disorders [33]. Although our epidemiological study does not explore causality or even pathophysiology, it supports these findings from basic research. Tinnitus burden correlated positively with the sum scores of the PHQ-9, GAD-7, and SSS-8. This correlation of symptom intensity supports the epidemiological association of a significantly higher prevalence of psychological symptoms in tinnitus participants. Furthermore, the logistic regression model showed that depression, anxiety, and somatic symptom disorder all had an independent influence on the occurrence of tinnitus, with somatic symptom disorders having the strongest effect. Hearing impairment itself was a significant risk factor for the occurrence of tinnitus. This did not change when adjusting for depression, anxiety, or somatization or for all three factors combined. Although previous literature suggests an association between hearing loss and depression, we did not observe hearing loss to influence the relationship of tinnitus and psychological disorders [34].

### Strengths and Limitations

The main strength of this study is its population-based design, which enables us to measure prevalences and the association of tinnitus with psychological disorders in the general population. 

However, this study contains two limitations, which are discussed below. Depression, anxiety, and somatic symptom disorders were assessed by a self-administered questionnaire. A high sum score on these questionnaires does not necessarily imply a confirmed clinical diagnosis, as this must always be based on a structured clinical assessment [12]. Furthermore, the causality of the association between tinnitus, depression, anxiety, and somatic symptom disorders remains unclear. In addition, the study cohort was drawn at random from the registrar’s office and participants were invited to the study site. Since participation was voluntary, we must assume some selection bias of unknown magnitude. Furthermore, a non-responder analysis is planned and will provide additional information on reasons for the loss to follow-up in future analyses [19].

## 5. Conclusions

This study shows that tinnitus correlates with symptoms of depression, anxiety, and somatization. Clinicians should be aware of this and assist patients in receiving comprehensive care. Tinnitus patients should be actively screened for symptoms of depression, anxiety, or somatization. Involving psychotherapists or specialists in psychosomatic medicine into the patient’s care at the first visit avoids many unnecessary consultations. Repeated medical consultations could increase the distress of tinnitus and lead to a more difficult treatment [35]. Since depression, anxiety, and somatic symptom disorders are more common in females, they appear to be a particularly vulnerable patient group. Future studies should aim to better understand the causality between psychological distress and tinnitus in order to improve treatment options and support interdisciplinary care. Prospective, population-based cohorts with a longitudinal follow-up over a longer time-span could assess the chronological order of onset of tinnitus, hearing loss, and psychological comorbidities.

## Figures and Tables

**Table 1:** Demographics and prevalence of tinnitus, depression, anxiety, and somatization.

	All (*n*)	Male (*n*)	Female (*n*)	*p*-Value
*n*	8539	4362	4177	<0.0001
Average Age years (SD)	60.7 (13.7)	61.4 (13.7)	59.9 (13.5)	<0.0001
Tinnitus prevalence % (*n*)	28.0% (2387)	31.7% (1.382)	24.1% (1.005)	<0.0001
Tinnitus burden 1–6 (SD)	2.39 (1.33)	2.35 (1.33)	2.44 (1.34)	0.11
PHQ-9 median score (Q1/Q3) *	2 (1/5)	2 (0/4)	3 (1/5)	<0.0001
PHQ-9 ≥ 10% (*n*)	5.5% (466)	4.4% (191)	6.7% (275)	<0.0001
GAD-7 median score (Q1/Q3) *	1 (0/4)	1 (0/3)	2 (0/5)	<0.0001
GAD-7 ≥ 10% (*n*)	3.9% (169)	2.6% (57)	5.3% (112)	<0.0001
SSS-8 mean score (SD)	5.70 (4.78)	5.03 (4.35)	6.40 (5.09)	<0.0001
SSS-8 ≥ 8% (*n*)	30.6% (2584)	25.0% (1072)	36.6% (1512)	<0.0001

SD: Standard deviation; Q1: 25th percentile; Q3: 75th percentile. * for |skewness| > 1, median score (Q1/Q3) is reported.

**Table 2:** Prevalence of depression, anxiety, and somatic symptom disorder among participants with and without tinnitus.

Tinnitus	Yes [95% CI]	No [95% CI]
Study cohort:		
PHQ-9 ≥ 10	7.9% [6.9%; 9.1%]	4.6% [4.1%; 5,2%]
GAD-7 ≥ 10	5.4% [4.3%; 6.9%]	3.3% [2.7%; 4.0%]
SSS-8 ≥ 8	40.4% [38.4%; 42.4%]	26.9% [25.8%; 28.0%]
European standard population 2013 *:		
PHQ-9 ≥ 10	9.5% [8.3%; 10.9%]	5.6% [5.0%; 6.2%]
GAD-7 ≥ 10	8.8% [7.6%; 10.1%]	5.5% [4.9%; 6.1%]
SSS-8 ≥ 8	41.0% [38.9%; 43.1%]	26.6% [25.5%; 27.7%]
German standard population 2021 *:		
PHQ-9 ≥ 10	9.3% [8.1%; 10.6%]	5.5% [5.0%; 6.1%]
GAD-7 ≥ 10	5.6% [4.3%; 7.3%]	3.3% [2.7%; 4.2%]
SSS-8 ≥ 8	41.6% [39.5%; 43.7%]	27.1% [26.0%; 28.2%]

* Results weighed to a reference population representative for the age structure of the European population in 2013 and the German population in 2021.

**Table 3:** Hearing impairment (WHO classification, better ear average across 0.5/1/2/4 kHz in dB).

WHO Hearing Impairment	All (*n*)	Males (*n*)	Females (*n*)	*p*-Value
No (<20 dB)	46.6% (2418)	41.8% (1111)	51.7% (1307)	<0.0001
Mild to moderately severe (20–64.9 dB)	51.4% (2664)	56.0% (1490)	46.5% (1174)	<0.0001
Severe to complete (≥65 dB)	2.0% (104)	2.2% (58)	1.8% (46)	0.37

**Table 4:** Prevalence of hearing impairment (WHO classification, better ear average across 0.5/1/2/4 kHz in dB) among participants with and without tinnitus.

Tinnitus	Yes [95% CI]	No [95% CI]
Study cohort:		
No (<20 dB)	36.4% [33.8%; 39.1%]	50.2% [48.6%; 51.8%]
Mild to moderately severe (20–64.9 dB)	59.3% [56.6%; 62.0%]	48.6% [47.0%; 50.2%]
Severe to complete (≥65 dB)	4.3% [3.3%; 5.5%]	1.2% [0.9%; 1.6%]
European standard population 2013 *:		
No (<20 dB)	51.3% [48.4%; 54.2%]	66.8% [65.3%; 68.3%]
Mild to moderately severe (20–64.9 dB)	45.8% [42.9%; 48.7%]	32.5% [31.0%; 34.0%]
Severe to complete (≥65 dB)	2.9% [2.0%; 4.0%]	0.7% [0.5%; 1.0%]
German standard population 2021 *:		
No (<20 dB)	49.0% [46.1%; 51.9%]	63.9% [62.3%; 65.4%]
Mild to moderately severe (20–64.9 dB)	47.6% [44.8%; 50.5%]	35.2% [33.7%; 36.8%]
Severe to complete (≥65 dB)	3.4% [2.4%; 4.6%]	0.9% [0.6%; 1.2%]

* Results weighed to a reference population representative for the age structure of the European population in 2013 and the German population in 2021.

**Table 5:** Multiple logistic regression model on tinnitus, hearing impairment, and depression/anxiety/somatization.

Tinnitus (Yes)	OR	95% CI	*p*-Value
Mild to moderately severe hearing loss (vs. normal hearing), adjusted for:			
PHQ-9 ≥ 10	1.645	1.400, 1.934	<0.0001
GAD-7 ≥ 10	1.739	1.392, 2.178	<0.0001
SSS-8 ≥ 8	1.616	1.373, 1.904	<0.0001
PHQ-9 ≥ 10, GAD-7 ≥ 10, and SSS-8 ≥ 8	1.733	1.384, 2.174	<0.0001
Severe to complete hearing loss (vs. normal hearing), adjusted for:			
PHQ-9 ≥ 10	4.677	3.057, 7.186	<0.0001
GAD-7 ≥ 10	5.112	2.948, 8.914	<0.0001
SSS-8 ≥ 8	4.502	2.932, 6.943	<0.0001
PHQ-9 ≥ 10, GAD-7 ≥ 10, and SSS-8 ≥ 8	4.819	2.763, 8.445	<0.0001

OR: Odds ratio; CI: Confidence interval.

## Data Availability

Not applicable.
